# The emergence of azithromycin-resistant *Salmonella* Typhi in Nepal

**DOI:** 10.1093/jacamr/dlaa109

**Published:** 2020-12-21

**Authors:** Pham Thanh Duy, Sabina Dongol, Abhishek Giri, Nguyen Thi Nguyen To, Ho Ngoc Dan Thanh, Nguyen Pham Nhu Quynh, Pham Duc Trung, Guy E Thwaites, Buddha Basnyat, Stephen Baker, Maia A Rabaa, Abhilasha Karkey

**Affiliations:** 1 The Hospital for Tropical Diseases, Wellcome Trust Major Overseas Programme, Oxford University Clinical Research Unit, Ho Chi Minh City, Vietnam; 2 Centre for Tropical Medicine and Global Health, Nuffield Department of Medicine, University of Oxford, Oxford, UK; 3 Oxford University Clinical Research Unit, Patan Academy of Health Sciences, Kathmandu, Nepal; 4 Patan Hospital, Lalitpur, Kathmandu, Nepal; 5 Cambridge Institute of Therapeutic Immunology & Infectious Disease (CITIID), Department of Medicine, University of Cambridge, Cambridge, UK

## Abstract

**Background:**

Typhoid fever remains a significant cause of morbidity and mortality in Asia and Africa. The emergence of azithromycin resistance in South Asia is concerning, as azithromycin is one of the last effective oral drugs for treating typhoid.

**Objectives:**

To describe the molecular mechanism and phylogenetics of azithromycin-resistant (Azith^R^) *Salmonella* Typhi isolates from Patan Hospital, Kathmandu, Nepal.

**Methods:**

Whole-genome sequences of three Azith^R^  *S*. Typhi isolates (MIC >256 mg/L) were analysed and compared with a global collection to investigate the azithromycin resistance mechanism and phylogenetic structure. Clinical information is reported for one of the three patients infected with Azith^R^  *S*. Typhi.

**Results:**

The three Azith^R^ isolates belonged to the H58 lineage and were genetically identical; they were distantly related to contemporaneous *S*. Typhi from Nepal and Azith^R^  *S*. Typhi recently described in Bangladesh. Azithromycin resistance was mediated by a non-synonymous mutation in the *acrB* gene (R717L). The three Azith^R^ isolates showed reduced susceptibility to ciprofloxacin (double mutation in the *gyrA*: S83F and D87G), and were susceptible to ampicillin, chloramphenicol and co-trimoxazole. Clinical information from one patient suggested non-response to azithromycin treatment.

**Conclusions:**

This is the first molecular description of Azith^R^  *S*. Typhi in Nepal. These organisms showed no phylogenetic link to Azith^R^  *S*. Typhi in Bangladesh. Our data suggest that increasing use of azithromycin may pose a strong selective pressure driving the emergence of Azith^R^  *S*. Typhi in South Asia. Further investigations are needed to evaluate treatment responses to azithromycin, predict evolutionary trajectories, and track the transmission of these organisms.

## Introduction

Typhoid fever is a life-threatening systemic infection predominantly caused by *Salmonella enterica* serovar Typhi (*S.* Typhi). Although the disease has been controlled in developed countries, it continues to cause significant morbidity and mortality in resource-poor settings in Asia and Africa. Effective antimicrobial therapy is essential to avoid deaths and serious complications. However, *S.* Typhi has continually evolved resistance to antimicrobials used for its treatment, posing a constant clinical challenge and likely exacerbating disease burden.^1^ Multi-drug resistance (MDR; resistance to chloramphenicol, ampicillin, trimethoprim/sulfamethoxazole) first evolved in *S.* Typhi in the late 1980s, followed by fluoroquinolone resistance in the 1990s.^2^ Third-generation cephalosporins have since been used for typhoid treatment, but the emergence of extensively-drug resistant (XDR; MDR plus resistance to fluoroquinolones and third-generation cephalosporins) *S*. Typhi in Pakistan^3^ has reduced the clinical efficacy of these drugs and raises concerns regarding the imminent spread of untreatable *S*. Typhi.

Azithromycin is effectively the last remaining oral antimicrobial to treat typhoid fever and has been widely used for empirical therapy in South Asia.^4^ Although azithromycin resistance in *S*. Typhi has rarely been reported, an increasing reliance on this drug has led to the emergence of azithromycin-resistant (Azith^R^) *S*. Typhi in South Asia. A recent study in Bangladesh indicated that azithromycin resistance (MIC >32 mg/L) in *S.* Typhi was associated with a non-synonymous mutation (R717Q) in the *acrB* gene, which encodes an efflux pump.^5^ There are limited data on clinical responses to azithromycin in Azith^R^  *S*. Typhi-infected patients. Here, we report the genomics, antimicrobial resistance profiles, and phylogenetic relatedness of three Azith^R^  *S*. Typhi isolates obtained from typhoid fever outpatients visiting a hospital in Nepal. We report clinical manifestations and azithromycin response data for one of the patients.

## Materials and methods

Patan Hospital (Kathmandu, Nepal) serves ∼320 000 outpatients and ∼20 000 inpatients annually. Typhoid fever is frequently managed in the outpatient department (OPD) of the hospital and blood culture is routinely performed when enteric fever is suspected.^6^ Antimicrobial susceptibility testing is performed by a modified Bauer-Kirby disc diffusion, with Etests^®^ to determine MICs (bioMérieux, France); results are interpreted using CLSI guidelines.^7^ In August and September 2019, the microbiology department identified three patients with Azith^R^  *S*. Typhi attending the OPD. Clinical information was only available for one of these three patients infected with Azith^R^  *S*. Typhi.

Total genomic DNA from *S*. Typhi isolates [including one contemporaneous azithromycin-susceptible (Azith^S^) isolate] was extracted and whole genome sequencing was performed on an Illumina MiSeq to generate 250 bp paired-end reads (raw data deposited in ENA, project PRJEB37899). Sequence data from this study were combined with 1508 *S*. Typhi H58 genomes published previously between 2015 and 2019 (Table S1, available as Supplementary data at *JAC-AMR* online).^3^^,^^5^^,^^8–12^ Single nucleotide polymorphisms (SNPs) were called using previously described methods.^12^ Briefly, raw reads were mapped to the reference sequence of *S*. Typhi strain CT18 (accession no: AL513382), plasmid pHCM1 (AL513383) and pHCM2 (AL513384) using SMALT (version 0.7.4) (http://www.sanger.ac.uk/resources/software/smalt/). SNPs were called against the reference sequence and filtered using SAMtools.^13^ The allele at each locus in each isolate was determined by reference to the consensus base in that genome using SAMtools mpileup and removal of low confidence alleles with consensus base quality ≤20, read depth ≤5 or a heterozygous base call. SNPs in phage regions, repetitive sequences or recombinant regions were excluded, which resulted in a final core SNP alignment with a total length of 3326. SNPs were subsequently annotated using the parseSNPTable.py script in the RedDog pipeline (https://github. com/katholt/RedDog). A subset of 68 SNPs were used to assign *S*. Typhi isolates to previously defined lineages according to the existing extended *S*. Typhi genotyping framework.^11^ SeaView (http://doua.prabi.fr/software/seaview) was used to visualize the SNP alignment and identify the SNP distance between the Nepali Azith^R^  *S*. Typhi isolates and Azith^S^ isolate. A maximum likelihood phylogeny was inferred from the above SNP alignment using RAxML (v8.2.8)^14^ with the generalized time-reversible model and a Gamma distribution to model the site-specific rate variation (GTR+Г). Support for the maximum likelihood tree was assessed via bootstrap analysis with 100 pseudo-replicates. The phylogeny was visualized using ITOL.^15^ Antimicrobial resistance (AMR) genes and plasmid contents were determined using SRST2 with default settings,^16^ with ARG-Annot database^17^ and Plasmidfinder^18^ used as respective reference databases.

### Ethics

All patient data were fully anonymized. The study was conducted in accordance with the guidelines of the Patan Hospital, Kathmandu, Nepal. The Nepal Health Research Council waived ethics review for this study.

## Results

On 25 August 2019, a 28-year-old male from Nakkhu (Lalitpur District) presented to the OPD following four days of anorexia and persistent fever despite three days of paracetamol use. General and systemic examinations were normal except for a fever of 38.9°C on presentation. Investigations revealed a haemoglobin count of 13.7 g/dL, total white blood cell count of 8.6 × 10^3^/μL with increased neutrophils (DLC: N-83, L-17), platelet count of 195 × 10^3^/μL, and C-reactive protein of 18.7 mg/dL. Urine microscopy and analysis were normal and blood culture was performed. A clinical diagnosis of enteric fever was made and oral azithromycin (1 g once/day) was administered. The patient was asked to return for the blood culture reports after 72 h. On day two of culture, his blood culture was positive for *S.* Typhi, which was found to be Azith^R^ (6 mm zone of inhibition on disc diffusion, MIC >256 mg/L). The patient did not return for the scheduled 72 h follow-up, but was traced on day seven of treatment. On day seven, the patient reported a fever of 38.3°C lasting for two days. Physical examination showed no abnormalities, but laboratory examinations were repeated due to the previous blood culture results. Repeat laboratory analysis showed a haemoglobin count of 13.5 g/dL, total white blood cell count of 6.9 × 10^3^/μL, continued increased neutrophils (DLC: N-77, L-23), platelet count of 219 × 10^3^/μL, and C-reactive protein of 23 mg/dL. A repeat blood culture was performed and an Azith^R^  *S.* Typhi was again isolated. The patient was admitted and administered intravenous ceftriaxone. The patient became afebrile after 48 h of ceftriaxone treatment and was discharged after seven days of intravenous ceftriaxone. Two additional Azith^R^  *S.* Typhi isolates displaying identical resistance phenotypes were identified from a 53-year-old female from Nakkhu, Lalitpur (25 August 2019) and a 26-year-old male from Ramshatol, Ramechhap (9 September 2019); no clinical information was available for these patients. No epidemiological links were suspected between these three patients.

**Figure 1.**
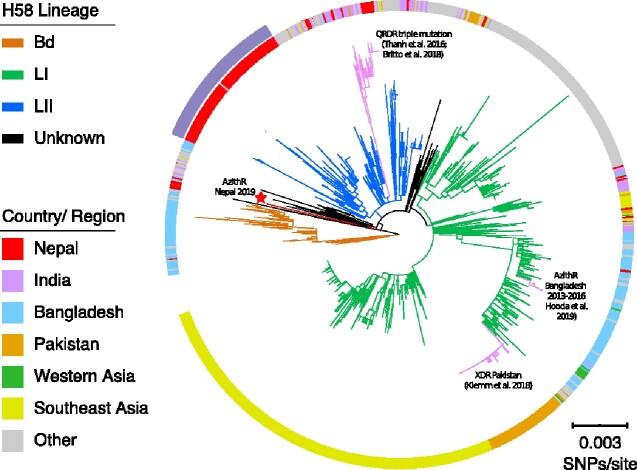
Phylogenetic relationships between azithromycin-resistant Nepali H58 isolates and global H58 isolates. Rooted maximum likelihood tree (CT18 was used as an outgroup to root the tree and pruned for visualization) reconstructed based on the SNPs of 1512 H58 isolates. Branches indicating three major H58 lineages are coloured in orange (lineage Bd), green (lineage I), and blue (lineage II). The red terminal branch (also highlighted with a red star) shows the three azithromycin-resistant isolates described in this study. Branches highlighted in pink show different clusters associated with fluoroquinolone resistance, extensive drug resistance, and azithromycin resistance reported recently in Nepal, Pakistan and Bangladesh, respectively. The ring around the phylogeny indicates the location from which each isolate originates. The outside purple arc indicates the Nepali cluster containing most contemporaneous Nepali *S*. Typhi H58 isolates. The scale bar indicates 0.003 SNPS/site. QRDR, quinolone resistance-determining regions.

Genomic and phylogenetic analyses showed that these Azith^R^  *S*. Typhi isolates and the contemporaneous Azith^S^ isolate from this study belonged to the H58 lineage. The three Azith^R^ isolates were genetically identical and differed from the Azith^S^ isolate by 42 SNPs. A previous study showed that most contemporaneous Nepali H58 isolates formed a separate cluster nested within H58 lineage II,^10^ including the Azith^S^ isolate from this study (Figure 1); however, the three Nepali Azith^R^ isolates did not cluster within the Nepali cluster, and instead exhibited a phylogenetic distance of about 27 SNPs relative to this cluster. Further, the three Nepali Azith^R^ isolates were also distantly related to all other H58 isolates from the global collection (Figure 1), and were not linked to the Azith^R^ H58 isolates recently reported in Bangladesh, which belonged to H58 lineage I.^5^

Regarding AMR, none of the Nepali Azith^R^ H58 isolates carried an acquired AMR gene, but instead harboured a non-synonymous mutation in the *acrB* gene (STY0519), changing arginine (R) to leucine (L) at codon 717. R717L and R717Q mutations in *acrB* have been found to confer Azith^R^ in Bangladeshi *S*. Paratyphi A and *S*. Typhi, respectively.^5^ Furthermore, the Nepali Azith^R^ H58 isolates exhibited double mutations in the *gyrA* gene (S83F, D87G), leading to reduced fluoroquinolone susceptibility; no *parC* mutations were observed.

## Discussion

The emergence of Azith^R^  *S*. Typhi warrants further detailed clinical investigation to better understand the correlation between *in vitro* azithromycin susceptibility and clinical responses to azithromycin treatment. *In vitro* resistance to azithromycin does not agree with *in vivo* effectiveness because the drug is mostly concentrated intracellularly.^19^ A previous study found no difference in response to azithromycin treatment among patients infected with *S*. Typhi exhibiting azithromycin MICs from 4–16 mg/L.^20^ However, *S*. Typhi isolates with azithromycin MICs >16 mg/L are rare and clinical data on treatment responses in such organisms are lacking. Here, we report three patients infected with Azith^R^  *S*. Typhi with MIC >256 mg/L, one of whom was given oral azithromycin 1 g once/day. Although patient adherence to initial treatment cannot be ensured, clinical investigation suggests that the patient might not have adequately responded to azithromycin treatment and experienced microbiological failure. Further clinical and epidemiological investigations are needed to examine the increase of azithromycin resistance in *S*. Typhi in South Asia, limit its transmission, and improve empirical antimicrobial regimens.

Our data show that azithromycin resistance mutations at codon 717 (*acrB* gene) have independently emerged across distantly related H58 lineages in Nepal and Bangladesh, suggesting that increasing use of azithromycin for treating typhoid fever may impose a strong selective pressure driving the emergence and spread of Azith^R^  *S*. Typhi. Convergent evolution towards azithromycin resistance in *S*. Typhi is of particular concern, as the development of azithromycin resistance mutations in XDR *S*. Typhi will eventually result in potentially untreatable infections. Further studies are needed to understand the driving forces and fitness effects of such resistance mutations.

All three Azith^R^  *S*. Typhi isolates in this study were genetically identical, phylogenetically unrelated to all contemporary Nepali *S*. Typhi, and exhibited double mutations in the *gyrA* gene. We hypothesize these patients may have been infected from the same source. Additionally, the infecting organisms originate from an H58 variant that has been replaced by the dominant H58 lineage II.^10^ This observation suggests this variant may be circulating at a low prevalence in the human population or has been maintained in a reservoir separate from the environmental transmission cycle, such as through asymptomatic carriage in the gallbladder.

In conclusion, we report three outpatients infected with highly azithromycin-resistant (MIC >256 mg/L) *S*. Typhi in Nepal. One patient was given oral azithromycin 1 g once/day and experienced prolonged fever until rescue treatment with ceftriaxone. All three Azith^R^ isolates were genetically identical H58 variants that were phylogenetically distinct from other contemporaneous Nepali *S*. Typhi. Azithromycin resistance was mediated by a chromosomal mutation R717L in the *arcB* gene. Further clinical and epidemiological investigations are required to track their transmission and evaluate clinical responses in patients infected with these important pathogens.

## Supplementary Material

dlaa109_Supplementary_Data
